# Randomized Controlled Trial on the Effect of *Channa striatus* Extract on Measurement of the Uterus, Pulsatility Index, Resistive Index of Uterine Artery and Superficial Skin Wound Artery in Post Lower Segment Caesarean Section Women

**DOI:** 10.1371/journal.pone.0133514

**Published:** 2015-07-29

**Authors:** Mohd Rizal Abu Bakar, Azidah Abdul Kadir, Siti Zubaidah Abdul Wahab, Ahmad Helmy Abdul Karim, Nik Hazlina Nik Hussain, Norhayati Mohd Noor, Julia Omar, Saringat Bin Bai @ Bae, Wan Haslindawani Wan Mahmood, Asrenee Abdul Razak, Rohaizan Yunus

**Affiliations:** 1 Advanced Medical and Dental Institute, Universiti Sains Malaysia, Kubang Kerian, Malaysia; 2 School of Medical Sciences, Universiti Sains Malaysia, Kubang Kerian, Malaysia; 3 School of Pharmaceutical Sciences, Universiti Sains Malaysia, Kubang Kerian, Malaysia; 4 International Medical University, Seremban, Malaysia; St Francis Hospital, UNITED STATES

## Abstract

**Aim:**

To compare the mean of anteroposterior (AP) measurements of the uterus in longitudinal and oblique transverse planes, and the pulsatility index (PI) and resistive index (RI) of the uterine artery and superficial skin wound artery between patients taking *Channa striatus* and placebo.

**Background:**

*Channa striatus*, also known as haruan, is a fresh water snakehead fish consumed in many parts of Southeast Asia. *Channa striatus* is also normally consumed by women postpartum to promote wound healing as well as to reduce post-operative pain.

**Methodology:**

This study is a randomised, double blind, placebo-controlled study conducted in women after Lower Segment Caesarean Section (LSCS). Subjects were randomised to either a *Channa striatus* or a placebo group and were given a daily dosage of 500 mg of *Channa striatus* extract or 500 mg maltodextrin, respectively, for six weeks post LSCS. The anteroposterior measurements of the uterus in the longitudinal and oblique transverse planes, and the pulsatility index (PI) and resistive index (RI) of the uterine and superficial skin wound arteries were assessed using pelvic Gray-scale ultrasound and Doppler ultrasound at baseline (Day 3) and at two weeks, four weeks and six weeks post-operatively.

**Results:**

Sixty-six subjects were randomised into the study with 33 in the *Channa striatus* group and 33 in the placebo group. No significant differences were detected in terms of the pulsatility index (PI) and the resistive index (RI) of the uterine and superficial skin wound arteries between the *Channa striatus* and placebo groups. However, in the *Channa striatus* group, the AP measurements of the uterus on the longitudinal and oblique transverse planes were significantly lower compared to the placebo group (p<0.05 and p<0.001, respectively).

**Conclusion:**

Daily intake of *Channa striatus* extract results in marked differences compared to placebo in terms of uterine involution and recovery in women post LSCS.

**Trial Registration:**

www.isrctn.com 11960786

## Introduction

*Channa striatus* is a fresh water snakehead fish well known in the Southeast Asian region for its protein and as a traditional remedy. Locals believe this fish is efficacious in treating wounds, relieving pain and boosting energy in the sick and elderly [1]. In addition, this fish is synonymous with use in a postnatal diet [2] and recuperation from illness or surgery [3]. In postpartum mothers, *Channa striatus* is consumed to accelerate the wound healing process [2,4].

Animal studies have shown that *Channa striatus* extract influences the different phases of wound healing by enhancing dermal wound healing [4,5]. It has also been proven to contain all the essential amino acids required for wound healing, particularly glycine and polyunsaturated fatty acids, which can promote prostaglandin synthesis [6,7]. High levels of these specific amino acids, fatty acids and arachidonic acid are effective in wound healing by promoting the initiation of a series of reactions: remodelling of collagen, re-epithelisation of the wound and induction of wound contraction [1,8,9].

Although extensive studies have been conducted on the biomedical properties of *Channa striatus*, most of these studies were performed on animals and limited clinical data in humans is available [10]. To our knowledge, there are no published reports on the effect of *Channa striatus* extract on uterus involution assessed by conventional or Doppler ultrasound in women post LSCS. In the present study, post LSCS women were chosen as the subjects because they are the main group of *Channa striatus* consumers in our population.

The use of ultrasound in this study allowed objective assessment of uterus involution during the puerperium. Moreover, it has been suggested that ultrasound is useful in assessing uterine scarring following LSCS [11]. We hypothesised that measurement of resistance flow indices, pulsatility index (PI) and resistive index (RI) from the flow velocity waveforms of the uterine artery postpartum would provide new information on the effect of *Channa striatus* extract on hemodynamic events during the postpartum involution process. Thus, the present study was designed to compare measurements of the uterus, PI and RI of the uterine artery and superficial skin wound artery between *Channa striatus* and placebo groups of women who had undergone LSCS.

## Materials and Methods

### Study design and setting

This study was a randomised, double blind, two-arm parallel comparative study of *Channa striatus* extract versus placebo. The study was conducted in women who had undergone LSCS at the Universiti Sains Malaysia Hospital (HUSM). The date range for patients recruitment and follow-up were from 15 March 2011 to 14 March January 2014.

### Study population

Inclusion criteria were women aged between 18 and 40 years who had undergone LSCS and who had no present active medical, surgical or gynaecological problems. The exclusion criteria were women who had taken any form of herbal extract in the previous three months prior to study entry or during the study period. Women with a history of drug or alcohol abuse, those taking fresh C*hanna striatus* during the study period, those on warfarin or heparin, and women who had been diagnosed with clinically relevant cardiovascular, gastrointestinal, hepatic, neurological, endocrine, haematological or connective tissue disease or another major systemic disease that would influence the interpretation of the results were also excluded. In addition, women with a medical disorder requiring steroid or immunosuppressive therapy, a chronic cough or other condition that may cause an increase in intra-abdominal pressure or any congenital anterior abdominal wall defects were not eligible for inclusion in the study.

### Data collection procedure

The orally administered freeze dried *Channa striatus* extract and placebo (maltodextrin) were prepared by a certified laboratory at the School of Pharmaceutical Sciences, Universiti Sains Malaysia. Both the freeze dried *Channa striatus* extract and placebo were available as 250 mg capsules. The treatment group was prescribed 500 mg (2 capsules) per day of freeze dried *Channa striatus* extract while the placebo group was given 500 mg (2 capsules) per day of maltodextrin. The women took the study medication for a total of 6 weeks starting from the three days after LSCS. Subject numbers were allocated strictly sequentially, as generated by a computer (blocks of four) that provided allocation of subject numbers in a ratio of 1:1 to either *Channa striatus* extract or placebo. Only two co-investigators who had prepared the product and a statistician knew the randomisation scheme. As the study was double blind, once a subject had been randomised, the study treatment was not known by either the subject or the investigators.

Written informed consent was obtained from the women 24–72 hours after elective or emergency LSCS. The researchers ensured that all women were fully conscious and comfortable before informed consent was taken. Information on demographic data and past and current medical history was obtained by interviewing the women.

On Day 3 post LSCS, the women underwent Gray-scale ultrasound and Doppler ultrasound pelvic examination. Day 3 was chosen for the ultrasound because wound inspection was done at this time and the abdominal bandage that covered the wound was removed. Then, the subjects were randomised to either the *Channa striatus* or placebo group. The ultrasounds were repeated at Week 2, Week 4 and Week 6 postoperatively. At each visit, any adverse event (AE) and concomitant medications were recorded. In addition, the women’s compliance was measured at each visit according to the number of capsules taken.

Assessment of liver function, renal function and full blood count were conducted at baseline and at the last visit (postoperative Week 6) to measure the safety profiles of *Channa striatus* and placebo. In this study, all the women underwent standard procedures for LSCS and received standard postoperative pain management.

### Outcome measures

The outcome measures used in this study were longitudinal and oblique transverse measurement of the uterus, and PI and RI of the uterine and superficial skin wound arteries. All ultrasound examinations were performed using the Acuson X300 ultrasound machine from Siemens, which was manufactured in 2011. The 3.5MHz and 7.5–13.5MHz ultrasound probes were used in this study. In order to ensure the most accurate data collection, only one co-researcher performed all the ultrasound examinations.

### Gray-scale ultrasound for uterine assessment

Ultrasound was performed using real time Acuson (X300), an ultrasound machine with a high resolution linear transducer of 3.5 MHz frequency. The patient is required to have a full bladder before the ultrasound of the uterus is performed. A gentle compression with the probe was performed and the measurements were made between uterine contractions. The uterus was assessed in two planes comprising the longitudinal and oblique transverse planes due to the anatomical position of the uterus, which cannot be evaluated in the true transverse plane in an ultrasound study. In the longitudinal and oblique transverse sections, the maximum anteroposterior (AP) diameter was measured with three readings in each. The average of these three readings was then calculated. This method of measurement was similar to that used in the study by Al-Bdour *et al*. 2004 [12].

### Doppler ultrasound for assessment of the uterine artery

The uterine artery is identified by colour Doppler, directing the transducer to the lateral wall of the uterus in the region of the lower uterine segment. The RI and PI measurements were taken at the point where the uterine artery crossed the external iliac artery. Either right or left uterine artery RI or PI values were recorded in this study. This is because the study by Van Schoubroeck *et al*. in 2006 showed there is no significant difference between right and left uterine artery RI and PI values [13]. Only single RI and PI measurements were taken once the best Doppler shift waveform was achieved.

### Doppler ultrasound for assessment of the superficial skin wound artery

The ultrasound was performed using the real time Acuson (X300), high end ultrasound machine with a high resolution linear transducer of 7.5–13.5 MHz frequency. The superficial LSCS skin wounds were identified and RI and PI of the superficial skin wound artery were measured. Only single RI and PI measurements were taken at a single site at the superficial LSCS skin wound once the best Doppler shift waveform was achieved.

### Statistical analysis

Sample size was calculated for all objectives of the study and the biggest sample size calculated was taken. The calculations were done using Power and Sample Size Calculation Software [14] for comparing two means between treatment group and placebo group. The biggest sample size calculated was based on the study by Nakai *et al*. on the RI of the superficial wound artery [15]. By using a standard deviation of 0.08, taking the power of 80%, detectable difference of 0.1 and level of significance of 0.05, the calculated sample size for each group was 26. However after considering of 30% drop-out, the sample size for each group is 34.

The study used a (modified) intention-to-treat (ITT) analysis approach where all subjects who took at least one dose of study drug and had at least one post-baseline efficacy evaluation are analysed based on their original randomisation. Subjects were omitted for analysis if they had no baseline data and/or were without at least one post-baseline evaluation. In case of missing data due to drop-outs, the data was imputed according to the Last Observation Carried Forward (LOCF) principle. Repeated measures for analysis of variance (ANOVA) were used to compare AP measurements of the uterus in longitudinal and oblique transverse planes, and the PI and RI of the uterine and superficial skin wound arteries throughout the 6 week intervention. Per protocol analysis was done to 49 subjects who completed all the study visits for comparison. Statistical analyses were performed using the Statistical package Social Sciences (SPSS) (PASW 20.0) under license of the Universiti Sains Malaysia.

### Ethics statement

The research proposal was approved by the Human Research Ethics Committee, Universiti Sains Malaysia (Reference number: USMKK / PPP/JEPeM [220.3(04)]. The date of approval was 31^st^ December 2009. Written informed consent was used in this study.

This trial is registered in the ISRCTN registry. The ISRCTN Registration number is 11960786. The registry website: www.**isrctn**.com/. The authors confirm that all ongoing and related trials for this drug/intervention are registered. The registration was done after completion of the study in order to fulfill the criteria for this journal. It is not a standard protocol or legislation in this country to register a clinical trial study for any trial registry.

## Results

**Fig 1:**
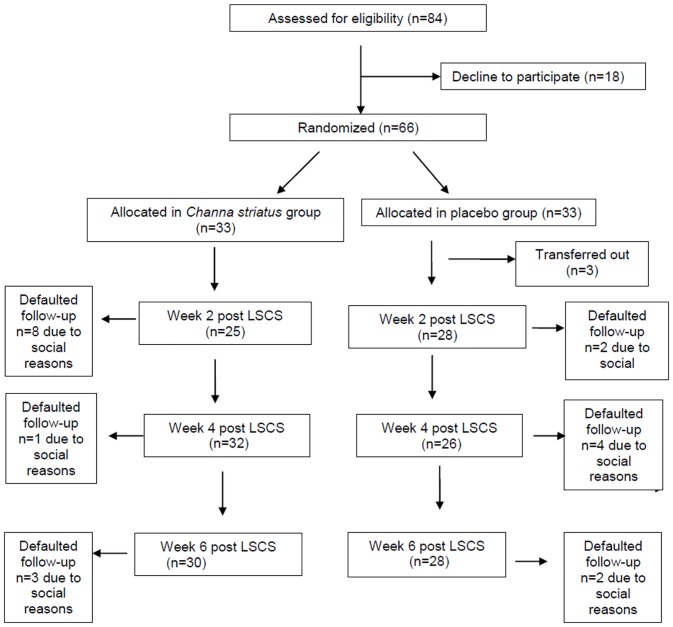
Flowchart of the study.

Eighty-four subjects consented to the study but 18 of these later refused to participate. Finally, sixty-six subjects were successfully randomised into the study. However, at post LSCS Day 3 another three patients were excluded from the study as they had been transferred out of the state (Fig 1). A total of 49 patients completed all the study visits i.e. 25 in *Channa striatus* and 24 in the placebo groups. The baseline characteristics of the all the subjects are presented in Table 1. There was no significant difference in baseline characteristics between the *Channa striatus* and placebo groups.

**Table 1:** Baseline characteristics of the subjects.

Demographic characteristics	*Channa striatus* (N = 33)		Placebo (N = 30)		p-value
	Mean(SD)	N (%)	Mean(SD)	N (%)	
Age	30.58(4.45)		28.10(5.56)		0.144*
Parity	2.52(1.72)		2. 20(1.69)		0.480*
Types of LSCS					
Elective		2(6.06)		2(6.67)	0.582**
Emergency		31(93.94)		28(93.33)	
Uterine AP measurement					
Longitudinal plane	73.73(6.30)		77.18(0.12)		0.316*
Oblique transverse plane	77.18(6.77)		68.92(21.56)		0.507*
Uterine artery RI	0.45(0.25)		0.42(0.16)		0.357*
Uterine artery PI	0.91(0.12)		0.73(0.24)		0.143*
Superficial skin artery RI	0.43(0.10)		0.42(0.15)		0.778*
Superficial skin artery PI	0.91(0.15)		0.76(0.25)		0.141*

*Determined by Independent-t test

**Determined by Simple logistic regression

LSCS, Lower Segment Caesarean Section; AP, anterior posterior; RI, resistive index; PI, pulsatility index.

Table 2 shows the group differences based on time for the longitudinal, oblique transverse plane of uterine AP measurements, and RI and PI measurements of the uterine and the superficial arteries for 63 subjects based on (ITT) analysis. Decreases were seen in AP measurements of the uterus from Day 3 to Week 6 post LSCS in both the *Channa striatus* and placebo groups. There were significant differences in the AP measurements of the uterus on the longitudinal plane and oblique transverse plane between the groups based on time throughout the study period (F = 3.93, p = 0.013). However, no significant differences were detected in terms of PI and RI of the uterine and superficial skin wound arteries between the *Channa striatus* and placebo groups.

**Table 2:** Between group differences based on time for longitudinal, oblique transverse planes of uterine AP measurements or RI and PI measurements of the uterine and superficial arteries (ITT analysis, N = 63).

Outcomes	Group	Day 3	Week 2	Week 4	Week 6	F-statistic	p-value
		Mean (95% CI)	Mean (95% CI)	Mean (95% CI)	Mean (95% CI)		
*Uterine AP measurement*							
Longitudinal	*C*.*striatus*	73.73	43.8	40.84	27.66	3.93(3)	0.013*
	(N = 33)	(68.32,79.13)	(36.58,51.06)	(36.80,44.89)	(23.76,31.55)		
	Placebo	69.77	56.05	46.57	38.02		
	(N = 30)	(64.10,75.43)	(48.45,50.81)	(42.32,50.81)	(33.93,42.10)		
Oblique Transverse plane	*C*.*striatus*	77.18	46.37	44.23	28.62	5.76(3)	0.002*
	(N = 33)	(71.73,82.63)	(38.84,53.89)	(39.79,48.66)	(24.73,32.51)		
	Placebo	68.92	53.13	48.46	38.51		
	(N = 30)	(63.20,74.63)	(49.24,65.02)	(43.81,53.11)	(34.43,42.59)		
Uterine artery RI	*C*.*striatus*	0.45	0.45	0.66	0.73	0.94(3)	0.423*
	(N = 33)	(0.40.0.49)	(0.38,0.53)	(0.61,0.72)	(0.65,0.81)		
	Placebo	0.42	0.51	0.62	0.74		
	(N = 30)	(0.37,0.46)	(0.43,0.59)	(0.56,0.67)	(0.65,0.82)		
Uterine artery PI	*C*.*striatus*	0.91	0.92	1.29	1.60	2.50(3)	0.068*
	(N = 33)	(0.84,0.97)	(0.77,1.07)	(1.81,1.41)	(1.43,1.77)		
	Placebo	0.73	1.03	1.31	1.62		
	(N = 30)	(0.67,0.80)	(0.87,1.19)	(1.19,1.42)	(1.43,1.80)		
Superficial skin artery RI	*C*.*striatus*	0.43	0.45	0.66	0.72	1.46(3)	0.232*
	(N = 33)	(0.38,0.47)	(0.37,0.52)	(0.60,0.71)	(0.64,0.80)		
	Placebo	0.42	0.50	0.58	0.71		
	(N = 30)	(0.37,0.47)	(0.42,0.58)	(0.52,0.64)	(0.63,0.79)		
Superficial skin artery PI	*C*.*striatus*	0.91	0.90	1.28	1.59	2.07(3)	0.113*
	(N = 33)	(0.84,0.98)	(0.75,1.04)	(1.16,1.39)	(1.41,1.77)		
	Placebo	0.76	1.04	1.46	1.52		
	(N = 30)	(0.69,0.84)	(0.89,1.19)	(1.06,1.85)	(1.34,1.17)		

AP, anterior posterior; RI, resistive index; PI, pulsatility index.

*Repeated measure ANOVA was applied followed by pairwise comparison with Bonferroni confidence interval adjustment. Assumption of normality, homogeneity of variances and compound symmetry were checked and fulfilled.

Table 3 shows the group differences based on time for the longitudinal, oblique transverse plane of uterine AP measurements, and RI and PI measurements of the uterine and the superficial arteries for 49 subjects that completed all the visits. There were significant differences in the AP measurements of the uterus on the longitudinal plane, oblique transverse plane and and PI of the uterine arteries between the groups based on time throughout the study period.

**Table 3:** Between group differences based on time for longitudinal, oblique transverse planes of uterine AP measurements or RI and PI measurements of the uterine and superficial arteries (per protocol analysis, N = 49).

Outcomes	Group	Day 3	Week 2	Week 4	Week 6	F-statistic	p-value
		Mean (95% CI)	Mean (95% CI)	Mean (95% CI)	Mean (95% CI)		
*Uterine AP measurement*							
Longitudinal	*C*.*striatus*	73.25	55.48	41.28	29.26	23.6(1)	<0.001*
	(N = 25)	(69.60, 76.90)	(52.58, 58.39)	(39.28, 43.28)	(26.84, 31.69)		
	Placebo	74.92	60.61	50.69	40.55		
	(N = 24)	(71.19, 78.65)	(57.65, 63.58)	(48.65, 52.74)	(38.08, 43.03)		
Oblique Transverse plane	*C*.*striatus*	76.99	58.94	44.32	30.74	10.9(1)	0.002*
	(N = 25)	(73.18, 80.80)	(56.17, 61.71)	(41.82, 46.81)	(28.43, 33.05)		
	Placebo	74.21	61.34	52.60	41.25		
	(N = 24)	(70.32, 78.10)	(58.51, 64.17)	(50.05, 55.14)	(38.89, 43.61)		
Uterine artery RI	*C*.*striatus*	0.44	0.58	0.69	0.81	1.4(1)	0.248*
	(N = 25)	(0.40, 0.48)	(0.55, 0.61)	(0.66, 0.71)	(0.78, 0.84)		
	Placebo	0.43	0.54	0.67	0.80		
	(N = 24)	(0.39, 0.47)	(0.51, 0.58)	(0.64, 0.70)	(0.77, 0.83)		
Uterine artery PI	*C*.*striatus*	0.91	1.18	1.35	1.78	5.2(1)	0.027*
	(N = 25)	(0.85, 0.96)	(1.11, 1.24)	(1.29, 1.41)	(1.72, 1.83)		
	Placebo	0.80	1.39	1.40	1.73		
	(N = 24)	(0.74, 0.85)	(1.03, 1.16)	(1.34, 1.45)	(1.68, 1.78)		
Superficial skin artery RI	*C*.*striatus*	0.42	0.57	0.68	0.80	1.6(1)	0.213*
	(n = 25)	(0.38, 0.46)	(0.54, 0.60)	(0.65, 0.71)	(0.77, 0.83)		
	Placebo	0.45	0.54	0.62	0.76		
	(N = 24)	(0.41, 0.50)	(0.51, 0.57)	(0.59, 0.66)	(0.73, 0.79)		
Superficial skin artery PI	*C*.*striatus*	0.90	1.14	1.33	1.76	3.0(1)	0.089*
	(N = 25)	(0.83, 0.96)	(1.09, 1.20)	(1.26, 1.40)	(1.67, 1.85)		
	Placebo	0.84	1.10	1.34	1.62		
	(N = 24)	(0.77, 0.90)	(1.04, 1.16)	(1.27, 1.41)	(1.53, 1.71)		

AP, anterior posterior; RI, resistive index; PI, pulsatility index.

*Repeated measure ANOVA was applied followed by pairwise comparison with Bonferroni confidence interval adjustment. Assumption of normality, homogeneity of variances and compound symmetry were checked and fulfilled.

Overall compliance with the study medication was 98%. No adverse effects (AEs) were reported and there were no changes in the safety profile parameters (full blood count, renal profile, and liver function test) before or after treatment.

## Discussion

In this study, we found that *Channa striatus* extract had a significant effect on uterine involution post LSCS compared to placebo. To our knowledge, this is the first study on this topic; therefore, we could not compare our results with those of with any other studies. Our data implied that *Channa striatus* is effective in facilitating uterine recovery in the postpartum period. A similar finding was also found in the study by Ho *et al*. on the effect of traditional Chinese herbal medicines on the process of uterus involution in women post LSCS and normal vaginal delivery [16]. In the study, ultrasound indices on 127 postpartum women and retrospectives questionnaire were used to query women about their frequencies of taking herbal medicines. They reported that consumption of *Eucommia ulmoides* negatively correlated with the AP diameter of the uterus and the cavity [16]. They speculate that increasing contractile activity of the uterus could explain the rapid uterus involution.

Uterine involution process in the puerperium involves massive remodelling of the extracellular matrix in association with cell proliferation and apoptosis [17]. Some of the remodeling events that occur naturally in the uterus, parturition, endometrial regeneration and uterine involution, have features in common with events of tissue injury and wound healing process, where they may occur after injury or in association with various pathologies [18].

We postulate that there are possible reasons for these findings. The effect of *Channa striatus* extract in rapid uterus involution rate could be due to an enhanced wound healing process and increased contractile activity of the uterus. As mentioned earlier, studies have shown that *Channa striatus* extract has wound healing properties [1,8,9]. Baie and Sheikh *et al*. conducted a study using *Channa striatus* extract cream to assess its wound healing effects in rats [8]. In this study, the researchers found that the *Channa striatus* extract enhanced the wound healing process by increasing the tensile strengths of the wound [8]. Rezqa and Syed Hassan in 2012 evaluated the effect of oral and topical *Channa striatus* on tensile strength, epithelisation, fibroblast count and hydroxyproline level in the healing of a laparotomy wound in malnourished rats [19, 20]. The results demonstrated that the group treated with oral and topical *Channa striatus* had significantly higher tensile strength and epithelial and fibroblast cell counts (P-value < 0.001).

We also postulate that the increased tensile strength may be attributed to the polypeptide formation produced by a combination of glycine with aspartic and glutamic acid in the presence of leucine, methionine, alanine and arginine [4]. The effectiveness of *Channa striatus* as a wound healing agent is also thought to be influenced by the high level of specific amino acids such as glycine and fatty acids such as arachidonic acid [8,9]. These two compounds are believed to be involved in the promotion of wound healing through the initiation of a series of mechanisms involving the remodelling of collagen, re-epithelialisation of the wound and induction of wound contraction [8,9,21, 22].

Prostaglandin has an important role in contraction of the smooth muscle of uterus [21]. As mention earlier, *Channa striatus* has high contents of arachidonic acid and polyunsaturated fatty acids that can promote prostaglandin synthesis [6,7]. It is postulated that the production of these prostaglandins promote uterine muscle contraction and rapid uterus involution. This effect was also seen in studies of the traditional Chinese herbal medicines [16,22,23]. The effect of the traditional Chinese herbal medicines called Sheng-Hua-Tang has been shown in animal models that it increases the myoelectric activity of rabbit uterine smooth muscle [22] and the contraction of the uterus when co-treated with oestrogen [23].

During pregnancy, the uterine vessels are dilated to increase the blood flow. There are many new vessel growths due to increase demand of blood flow from the fetus as well as the placenta. This physiological change will lead to the dilated, low resistance uteroplacental vessels with markedly increased blood flow. However, after the delivery, there are dramatic regression of the uterus, uterine arteries and their branches in the physiological involution process. Studies using Doppler ultrasound have been used to measure flow resistance indices of the uterine arteries in the puerperium [24,25]. The uterine artery can evaluate by direct visualization, i.e. examining the characteristics of the waveform, or by quantifying the PI and RI value. The PI (S-D/Mean) and the RI (S-D/S) are two semi-quantitative, angle—independent indices, where S is the maximum and D is the minimum of the Doppler shift frequency through the cardiac cycle. In pregnancy, there is a high vascularity and it is expected that the RI and PI would be lower compare to puerperium or non-pregnant state. The duration of physiological returns of the hemodynamic changes to non-pregnant state is a controversial issue. Study by Tekay and Japilla showed that the values were not reached even three months after delivery [25].

We noted an increasing trend of the readings of the RI and PI from Day 3 until Week 6 postpartum. The increase in the RI and PI values reflects the healing process in both groups. These findings were similar to those of other studies conducted by Mulic—Lutvica *et al*. [24] *and* Tekay and Jouppila *et al*. [25], which assessed the peripheral vascular resistance of the uterine artery in postpartum women. These studies found that the PI and RI values increased significantly from Day 1 until day 56 postpartum [24,25]. Our study had showed that there was an increasing trend of the values but however these values were still lower compare to values reported for non-pregnant women, which suggest a slow return of vascular physiology to a non-pregnant state [24, 25].

In the present study, no significant differences were found in the RI and PI of the uterine and superficial skin arteries between the *Channa striatus* and placebo groups in the ITT analysis. However, interestingly, the per protocol analysis showed there was significant difference between the groups for PI of the uterine artery. The findings from Doppler velocimetric analysis are inconclusive. This findings explain the true biological effect of healing process by *Channa striatus*. In puerperium, the physiological changes not only involves the muscle cells and decidua at the placental site but also the uterine arteries and their branches. However, the results of the ITT analysis would reflects the effect of pragmatic trial.

The *Channa striatus* extract was well tolerated in this study. There were no reported AEs and the overall compliance was good. Routine laboratory parameters remained within normal limits during the *Channa striatus* treatment, supporting the tolerability of this agent.

## Limitations and Recommendations

This study had several limitations. The major limitation was a high number of defaulters for the follow-up in this study. This was expected since it was difficult for the postpartum women to come to the follow-up due to time and social reasons. It is recommended that animal studies should be conducted to understand the mechanism of *Channa striatus* effect on uterine involution. Future works should also place greater emphasis on identifying bioactive compounds and research on standardisation of the extract.

## Conclusion

*Channa striatus* has a beneficial effect on uterine involution in post LSCS women and its use to enhance wound healing is highly recommended.

## Supporting Information

S1 CONSORT Checklist CONSORT checklist. (DOC)

S1 Dataset (SAV)

S1 Protocol Trial protocol. (DOCX)
