# Supplementary material for: Randomized Controlled Trial on the Effect of Channa striatus Extract on Measurement of the Uterus, Pulsatility Index, Resistive Index of Uterine Artery and Superficial Skin Wound Artery in Post Lower Segment Caesarean Section Women
Source: PLoS One. 2015 Jul 29;10(7):e0133514. doi: 10.1371/journal.pone.0133514 (PMC4519193; doi:10.1371/journal.pone.0133514)
Supplement: Supplementary file 1 [file pone.0133514.s003.docx]

**The effect of Channa striatus (ikan haruan) extract on post Lower segment caesarian Section (LSCS) women**

(a) **Research Background**

Channa striatus, a fresh water snakehead fish consumed in many parts of the Southeast Asian region, for centuries is known among local for protein and traditional remedy.^1^ It is believed to promote wound healing, as well as reduce post-operative pain.^2,3^ Haruan is the local name for *Channa striatus*. It is also known as *jalak* or *badus* in Sabah. It has been considered as a very good source of health food amongs Asians because it contains high levels of amino acids and fatty acids. *C. striatus* is normally consumed by women postpartum to promote wound healing.^3^

Scientific research carried out in Malaysia have unraveled the biomedical potential of the fish.^4^ The work carried out indicated that the fish *C. striatus* has a good anti pain property comparable to morphine.^5^ The anti-norciceptive properties make it suitable for reduction of post-operative pain.^3^ It also has anti- inflammatory, anti-microbial and anti-cancer property. ^6,7^

Haruan has been proved to influence the different phases of wound healing process. Channa striatus is reported to enhance dermal wound healing based on animal studies.^2,3^ Biochemical components such as amino acids and fatty acids are important for the synthesis of collagen fibers during wound healing. Arachidonic acid, a precursor of prostaglandin plays a vital role in healing the wounds. Haruan (*C. striatus*) contains all the essential amino acids for wound healing particularly glycine as well as high contents of arachidonic acid and polyunsaturated fatty acids that can promote prostaglandin synthesis.^7,8^ The bioactive compounds in *C. striatus* also acts synergistically on muscarinic, GABA and serotonergic receptor systems to produce the antinorciceptive effect.^8^ Thus It is postulated that it also have psychological effect in human through this pathways.

**Rationale of the study:**

Haruan is consumed by many Malaysians to promote healing in surgical wound especially women in the postpartum period. Although there are extenisve studies on beneficial of *C. striatus* biomedical properties, there studies were done on animal studies only and there is no clinical trial in human yet. So we undertake these study to get as scientific evidence to substantiate the claim. We decided to assess the effect of oral administered *C. striatus* extract that may have a possible role or potential in wound healing and reducing the post-operative pain in human. Post LSCS women were chosen for the study subjects because this group is usually the main reason for using Haruan in our population.

(b) **Objective (s) of the Research**

**General objective**

To evaluate the effectiveness of Channa striatus (ikan haruan) extract vs placebo (maltodextrin) on involution of uterus, healing of the LSCS wound, post-op pain, psychological effect and safety profile among postpartum LSCS women.

## **Specific objectives:**

To compare the difference between *Channa striatus* and placebo in terms of:

i. Post-operative pain score
ii. Wound healing based on:
    a. Wound Evaluation Scale (WES)
    b. Visual Analogue Scale (VAS)
    c. Patient’s satisfaction score (PSS)
iii. To compare mean anteroposterior measurement of the uterus in longitudinal and oblique transverse planes between patient taking *Channa striatus* (ikan haruan) and placebo (maltodextrin).
iv. To compare mean resistive index (RI) and pulsatility index (PI) of the uterine artery between patient taking *Channa striatus* (ikan haruan) and placebo (maltodextrin
v. To compare mean resistive index (RI) and pulsatility index (PI) and of the artery of the superficial skin wound between patient taking *Channa striatus* (ikan haruan) and placebo (maltodextrin).
vi. Safety profile based on Renal Function Test (RFT), Liver Function Test (LFT) and Full Blood Count (FBC).

**METHODOLOGY**

**Study Design**

This is a randomized, double blind, 2-arm parallel comparative study of *C. striatus* extract versus placebo (maltodextrin) among women who undergone LSCS.

**Source Population**

The source population will be patients for elective LSCS in Obstetric and Gynaecology Department, Universiti Sains Malaysia.

**Study Population**

The subjects will be recruited from Obstetric and Gynaecology Department Universiti Sains Malaysia (refer Figure 1). The following inclusion and exclusion criteria will be applied in this study:

Inclusion Criteria

a. Age ≥ 18 and ≤ 40 years
b. No present active medical, surgical and gynaecological problems
c. First LSCS (elective and emergency)

Exclusion Criteria

a. Taking any form of herbal extract in the last 3 months before study entry and during the study period
b. History of drug or alcohol abuse.
c. Taking fresh haruan during study period
d. Patient taking warfarin or heparin
e. Clinical relevant cardiovascular, gastrointestinal, hepatic, neurologic, endocrine, haematologic, connective tissue disease or other major systemic diseases that would influence the interpretation of results
f. Patients with medical disorder requiring steroid or immunosuppressive therapy with delay wound healing
g. Patient with chronic cough or other condition which may cause a rise in intra-abdominal pressure
h. Presence of any congenital anterior abdominal wall defects
i. Mental condition rendering the subject unable to understand the nature, scope and possible consequences of the study.
j. Evidence of uncooperative attitude, including poor compliance including inability to attend follow-up visit.

**Method of assigning subjects to treatment and placebo groups**

Subject eligibility will be established before treatment randomization. Subject number will be allocated strictly sequentially, as subjects are eligible for randomization. None of the investigators will know the randomization scheme. A randomization list will be generated using computer and will provide allocation of subject numbers in a ratio of 1:1 to either *C. striatus* extract versus placebo (maltodextrin).

**Blinding and procedures for breaking the blind**

This is double blind study and once a subject has been randomized, the study treatment that they will receive will not be known by both the subject and the investigator.

**Treatment compliance**

Subjects will be asked to return all unused medication. The number of capsules issued and minus the number of capsules returned will be used to calculate the capsules taken. From this information, compliance will be calculated.

Compliance =	Capsules taken during the period	x	100

Capsules which should have been taken

**Patient’s withdrawal-**

The investigator may cease study treatment and withdrew the subject or the subject may withdraw herself from participation in the study at any time. The reason for the withdrawal of a patient will be recorded in the CRF. Subject will be followed-up for a minimum of 30 days following the last dose of study drug.

Possible reasons for patient withdrawal include:

a. The need to take medication, which may interfere with study measurements.
b. Patient experiences an intolerable / unacceptable adverse event
c. Patient exhibits non-compliance with the protocol
d. Patient unwilling to proceed and / or consent is withdrawn
e. Investigator withdraws patient for reasons unrelated to the study drug (e.g. undercurrent illness)

**Investigational products**

The orally administered freeze dried *C. striatus* extract will be prepared in by a GMP-certified Laboratory, School of Pharmacy Universiti Sains Malaysia. Both the freeze dried *C. striatus* extract and maltodextrin will be available as capsule of 250 mg and will be packed by GMP Major Interest Sdn Bhd. Sungai Petani

**Doses and treatment regimens**

The treatment group will consume 500mg of freeze dried *C. striatus* extract while the placebo group will consume 500mg of maltodextrin. The medication will be taken orally, with water once daily. It may be taken at any time of the day, with or without a meal.

**Data Collection Procedure**

Written informed consent was obtained from the women 24-72 hours after the elective or emergency LSCS. The researchers ensured that the women were fully conscious and comfortable before informed consent was taken. Information on demographic data and past and concurrent medical history was obtained by interviewing the women.

During Visit 1 (day 3 post op), the subjects will be given questionnaires on demographic data and past medical and obstetric history and the Malay version EPDS to be filled. Then the investigator will performed physical examination (vital sign, cardiovascular and respiratory examination) on the subjects.

Pain will be assess by using Visual Analogue Pain Score to rate the extend of pain on Day-1, Day-2, Day-3, week 2, week 4 and week 6 post operative. All subjects will be instructed to take normal diet during the study period and will not be allowed to take any other herbal products orally or consumed *C. striatus*. The cosmetic assessments of the wound will be done by a Specialist who is part of the Clinical Trial Team. It will be done on Day-3, week 2, week 4 and week 6 post operation. Ultrasound of the pelvis and Doppler will be done on the subjects on Day-3 before discharge, week 2, week 4 and week 6 post operation. Subjects will be thoroughly examined by Medical specialists or Medical officers who are part of the Clinical Trial Team at every visit.

**STUDY MEASUREMENTS**

**1. Socio-demographic and medical data**

The following data will be collected in the case report form:

- Age, sex, race
- Significant medical and surgical history
- Type of anaesthesia for the LSCS
- Physical examination (height, weight and vital signs)
- Concomittant medication

**2. Ultrasound examination**

The ultrasound will be performed using real time Elagra (Siemens), high end ultrasound machine with high resolution linear transducer of 7.5 -13.5 MHz frequency. The patient needs to have a full bladder before the ultrasound of uterus is done. The patient in the supine position and the uterus will be assessed in two planes including longitudinal and transverse. The measurement will be done in longitudinal and transverse with three reading each. The average of these three reading will be calculated. The colour Doppler and/or power Doppler assessment of the incisional uterine section will be evaluated. The pulstility index (PI), resistive index (RI) and the percentage of the colour/ power intensity will be recorded.

**3. Healing of LSCS wound**

## Wound Evaluation Scale (WES) procedure

In supine position, the LSCS wound was exposed and examined based on WES. This data collected at post-op Day 3 and every subsequent visit.

*Tool:*

Wound Evaluation Scale ^13^

Wound healing were assessed by using Wound Evaluation Scale (WES). It consist of six items which were presence of wound discharge, edge inversion, step-off borders, contour irregularities, scar width more than 2mm and overall cosmesis (*see operational definition in Section 4.12)*. Each of these categories is graded on a 0 or 1 point scale. A total cosmetic score is derived by the addition of the scores of the six categorical variables.

WES was first developed by Holander *et al*., (1995a). In the study, WES is a part of a wound registry data sheet which consists of demographic characteristics, time from injury to evaluation, pertinent medical history, wound characteristics, type of anaesthesia, details of wound-cleansing methods, details of wound closure, and postoperative care. Hollander’s team found that the registry could be a reliable data collection instrument as it have substantial interobserver concordance for wound description (κ range, .55 to .97), wound infection (κ=1.0), and overall cosmetic appearance (~κ=.61). It is also easy to use and take less than one minute to complete. Singer (1998) also addressed that this scale was found to be reliable.

In 1998, Quinn *et al*., modified this this tool and compared with visual analogue cosmetic scale (VAS).They found that WES and VAS should be used together; because it is very useful, valid, reliable, and sensitive in detecting clinically important outcome measures for wound assessment in double blind randomized controlled trial.

For the item of scar’s width more than 2mm, the measurement was done by using flexible plastic measuring tape. This type of tapes holds highest validity and feasibility in measuring scar width at human’s abdomen with smallest standard error (Langemo *et al*., 1998; Fette, 2006). To measure the wound width in this study, the measuring tape was placed perpendicular to the wound length. Then, the width was measured by counting the pointing marks of the tape. One point means 1mm or 0.1cm (Figure 3.15.6). For non-linear scars, the investigator measured the width at their widest points, and calculated the means.

## Visual Analogue Scale (VAS) procedure

After WES done; while subject still in supine position, the cosmetic appearance of the wound was assessed by the investigator by using VAS. This data collected at post-op Day 3 and every subsequent visit.

*Tool:* Visual Analogue Scale ^14^

The original VAS consisted of a 100-mm line with "worst scar" at the right end of the line and "best scar" at the left end of the line. In this study, VAS ranged from 0 to 10cm. Using the line as a continuous entity, the investigator marked on the line where they thought the scar fit. The higher the score, the better the cosmetic appearance.

This VAS for cosmesis has been demonstrated to be reliable and valid. (Quinn *et al*., 1995). Quinn *et al*., 1995 demonstrated that the VAS showed good interobserver agreement with an Intraclass Correlation Coefficient of 0.75. Intraobserver agreement was also high across the four observers with Pearson correlation coefficients ranging from 0.73 to 0.87.This scale is widely used as it has good validity and reliability from previous studies (Crichton, 2001).

VAS has also been found to be highly sensitive and generally amenable to parametric statistical analyses (Quinn *et al*., 1995). Studies using these scales require relatively small sample sizes to demonstrate a significant difference between treatments. As a result, they are very powerful research tools. Duncan *et al*., 2006, Micominaco *et al*., 2009 and Fearmonti *et al*., 2010 also shown the visual analogue scale scar scoring and scar ranking methods to be consistent, reliable, valid, and feasible. However, when using photographs from camera for wound assessment, inconsistencies in patient’s position, camera angle and distance from patient can restrict their value in the assessment process (Thomas & Wysocki 1990). To reduce the discrement of cosmetic appearance of the wound in this study, the investigator rate the VAS of the wound on the spot during the wound insection at each visit.

## Patient’s Satisfaction Score (PSS) procedure

After VAS done, finally patient’s satisfaction about the appearance of the wound was asked to the subject by using Patient’s satisfaction Score (PSS). In this study, in order to assess the satisfaction score, a standard size of mirror (size A5) was given to the subjects to see their wound appearance while the wound was exposed at supine position, just after wound assessment done by the investigator. Then the patient verbalized their score (0 to 10).

*Tool*: Patient’s satisfaction score (PSS)^15^

Patient satisfaction was rated based on scoring of 0 to 10 with higher score indicating better satisfaction and lower score indicating poorer satisfaction. It has been validated by Coulthard *et al*., (2004), Blondeel *et al*., (2004) and revised by Amin *et al*., (2009).

**4. Post-op pain**

Subject were asked the intensity of post-operative pain by using Numeric Pain Rating Scale; collected at screening and every subsequent visit.

*Tool:* 0–10 Numeric Pain Rating Scale^16^

Post-op pain was rated based on Numeric Pain Rating Scale (NRS) of 0 to 10 with higher score indicating more severe pain and lower score indicationg less severe pain. This scale has been compared with Visual Analgue Scale (VAS) and Verbal Rating Scale (VRS) and several other pain scales for its validity and reliability to be used in measuring different types of pain (Huskisson, 1974,1983; Ohnhaus & Adler, 1975; Katz & Melzack, 1999). A systematic review in 2005 by Williamson & Hoggart addressed that NRS has good sensitivity compared to VAS and VRS. The acuracy of NRS was tested in 2007 by Krebs *et al*., which revealed that NRS have modest accuracy with 69% (95% CI 60-78) sensitivity.

**5. Safety measurements**

The safety evaluation will be determined by the incidence of adverse events and the definition of adverse events (AEs) and serious adverse events (SAEs) are as follows:

An adverse event (AE) is defined as any untoward medical occurrence (including clinically significant laboratory findings) in a patient or clinical investigation subject administered a pharmaceutical drug, and which did not necessarily have a causal relationship to the treatment. Adverse event may include:

- The significant worsening of the disease or symptoms of the disease under investigation following administration of the drug
- Any undercurrent illness with an onset after administration of the drug
- Exacerbation (i.e. increase in frequency or intensity) of a pre-existing condition or event

A serious adverse event (SAE) is defined as any untoward medical occurrence that at any dose of the investigational product, that fulfils one or more of the following criteria:

a. Results in death
b. Is life-threatening. A ‘ life-threatening’ adverse event refers to an event, which puts the patient at risk of death. It does not refer to an event, which hypothetically might cause death if it is more severe.
c. Requires in-patient hospitalization or prolongation of existing hospitalization. Hospitalization is defined as the patient being hospitalized overnight, or the patient’s hospital stay being prolonged for at least an additional overnight stay. Hospital admissions for elective surgery, for social reasons or for normal disease management procedures that are not the result of the worsening of an underlying condition will not be considered a serious adverse event.
d. Results in persistent or significant disability/incapacity
e. Is a congenital anomaly / birth defect
f. Is a malignancy
g. Is the result of an overdose? An important medical event, which jeopardizes the patient and may require medical or surgical intervention to prevent one of the above outcomes from occurring.

**Primary outcome measures**

1. Post-operative pain score

2. Wound healing based on:
2.1. Wound Evaluation Scale (WES)
2.2. Visual Analogue Scale (VAS)
2.3. Patient’s satisfaction score (PSS)

3. Safety profile based on Renal Function Test (RFT), Liver Function Test (LFT) and Full Blood Count (FBC)

4. Mean anteroposterior measurement of the uterus in longitudinal and oblique transverse planes
5. Mean resistive index (RI) and pulsatility index (PI) of the uterine artery
6. Mean resistive index (RI) and pulsatility index (PI) and of the artery of the superficial skin wound

Measured at baseline (day 3), two weeks, four weeks and six weeks post-operatively.

**DATA MANAGEMENT**

Case report forms (CRFs) will be provided for the recording of data. The forms will be in duplicate with carbonless paper. Data will be recorded legibly onto the record forms, preferably in ballpoint pen. If any data are not available, omissions will be indicated on the record forms. Correction should be made legibly and initialed. Correction fluid and covering labels must not be used.

**DETERMINATION OF SAMPLE SIZE AND STATISTICAL ANALYSES**

**1. Determination of sample size**

Sample size were calculated for each objectives. The calculations were done using Power and Sample Size Calculation Software (Dupont & Plummer, 1997) and the one that yielded the biggest sample size was taken as the study sample size.

Sample size for comparing two means between treatment group and placebo group were done. The parameters were as follows:

| α | = | Level of significance = 0.05 |
| --- | --- | --- |
| Power | = | 0.9 |
| σ | = | Standard deviation of the objective measurements [Pain, Wound Evaluation Scale (WES), Visual Analogue Scale (VAS) and Patient’s Satisfaction] |
| δ | = | Detectable difference in population means (clinically significant difference in objective measurements between *Channa striatus* extract group and placebo (maltodextrin) group based on literature review) |
| m | = | Ratio between placebo (maltodextrin) group and *Channa striatus* extract group = 1 |

The biggest sample size yielded by Objective ii. Based on previous study the detectable difference of VAS between control and treatment group was 15. The VAS measurement has a standard deviation of 19. The minimum required sample size was 35 and after considering the non-response rate of 20%, the sample size calculated for each group was 42. Finally, the estimated total sample size for both the treatment and placebo group was 84.

**2. Data entry and analyses**

Analyses will be done by using SPSS for windows version 12.0 and all numerical variables were expressed as Mean and standard deviation (SD) and categorical data as frequencies and percentage. Independent t-test will be used to analyzed numerical data such as age, weight, Body Mass Index (BMI ), systolic and diastolic blood pressure, EPDS score, clinical and ultra sound measurement of uterine size, patient’s satisfaction on wound appearence, skin aposition and pain score whereas chi-square test will be performed for the categorical data such as wound complication.

To detect the changes in all the variables throughout the six weeks period, ANCOVA will be conducted according to the group to which they were assigned. Summary changes are reported as means, standardizing changes for possible covariate imbalances between groups. All reported *p* values are 2-tailed. Cross sectional Time Series Multiple Logistic will be done. The controlled variables are- types of anaethesia and age.

**References:**

1. Wee KL: Snakeheads –their biology and culture. In Muir R, ed. Recent Advances in Aquaculture. Boulder, CO: Westview Press; 1982:181-213.

2. Mat Jais AM, McCullock R, Croft K. Fatty acid and amino acid composition in haruan as potential role in wound healing. Gen. Pharmacol 1994:25:947-950.

3. Mat Jais AM, Dambisya YM, Lee TL. Antinorciceptive activity of Channa Striatus (Haruan) extract in mice. J. Ethnopharmacol. 1997;57:125-130.

4.Mat Jais AM, Matori MF, Kittakoop P, Swanborirux K. Fatty acid composition in mucus and roe of Haruan, Channa striatus, for wound healing. Gen Pharmacol.1998;30:561-563.

5. Zakaria ZA, Mat Jais AM, Goh YM, Sulaiman MR and Somchit MN. Amino acid and fatty acid composition of an aqueous extract of Channa Striatus (Haruan) that exhibits antinorciceptive activity. Clinical and Exp. Pharmacol and Physiological .2007:34,198-204.

6. Michelle NYT, Shanthi G and Mohamad YL. Effects of orally administered Channa striatus extract against experimentally-induced osteoarthritis in rabbits. Intern J Appl Res Vet Med 2004;2(3);171-175.

7.Somchit MN, Solihah MH, Israf DA, Ahmad Z, Arifah AK and Mat Jais AM. Anti-inflammatory activity of Channa striatus, Channa micropeltes and Channa lucius extracts:chronic inflammatory modulation. J. Orient. Pharm. Exp. Med. 2004;4(2): 91-94

8. Zakaria ZA, Sulaiman MR, Mat Jais AM amd Somchit MN. Effect of various antagonists on Channa striatus fillet extract antinociception in mice. Can. J. Physiol. Pharmacol. 2005;83:635-642.

9. Cox JL, Holden JM and Sagovsky R. Detection of postnatal depression. Development of the 10-item Edinburgh Postnatal Depression Scale. British Journal of Psychiatry 1987;150:782-6.

10. Cox JL, Connor Y and Kendell RE. Prospective study of the psychiatric disorder of childbirth. British Journal of Psychiatry 1982;140:111–117.

11. Kadir AA, Nordin R, Ismail SB, Yaacob MJ and Mustapha WMRW. Validation of the Malay version of Edinburgh Postnatal Depression Scale. Asian Pacific Family Medicine Journal 2004; 3(1-2): 9-18.

12. Azidah AK, Shaiful BI, Rusli N & Jamil MY. Postnatal depression and socio-cultural practices among postnatal mothers in Kota Bahru, Kelantan, Malaysia. Medical Journal of Malaysia 2006;61(1): 76-83.

13. Hollander, J. E., Singer, A. J., Valentine, S. & Henry, M. C. (1995a). Wound Registry: Development and Validation. *Annals of Emergency Medicine,* **25(5),** 675-685.

14. Quinn, J. V., Drzewiecki, A. E. & Elmslie, T. J. (1995). Appearance scales to measure cosmetic outcomes of healed lacerations. *Am J Emerg Med,* **13,** 229-231.

15. Coulthard, P., Worthington, H. & Esposito, M. (2004). Tissue adhesives for closure of surgical incisions. *Cochrane Database Systematic Review,* **2**.

16. McCaffrey, M & Pasero, C. (1999). Pain: Clinical manual (2nd ed). Mosby, St Louis

**Benefit of study:**

To promote and commercialise Haruan *Channa striatus* product and unravelled the biomedical potential of the fish. Also as research plaftform to generate ideas for further studies regarding the medical use of this fish. This iniatives can help expedite higher value-capture in agricultural sector thus generate greater economic activities and lay foundation for a sustainable agro-based industry driven by agrobiotechnology development.

**Post op Day- 1**

Screening

Demographic data, physical examinations, EPDS evaluation

**Day- 3**	Ultrasound pelvis

Pain score

Randomize Subjects

Dispense Study Medication

**Placebo group**

**500 mg /day maltodextrin**

**Treatment group**

**500 mg /day of *C. striatus extract***

**Week 2**	Physical examinations

ultrasound pelvis, pain score

**Week 4** Physical examinations,Wound evaluations, ultrasound pelvis, pain score

**Week 6**	Physical examinations, Wound evaluations,ultrasound pelvis, pain score

#### Figure 1. Flow Chart Of The Study

**Appendix 1: CRF**

**DATE: FTIME: H ID NO: H**

**PROFORMA STUDY**

| (1) | Initial |  |
| --- | --- | --- |
| (2) | Age |  |
| (3) | Address |  |
| (4) | No Tel |  |
| (5) | Ethnicity |  |
| (6) | Gravida & Para |  |
| (7) | Indication for LSCS |  |
| (8) | Type of anaesthesia |  |
| (9) | Data for LSCS |  |

| **SCREENING INCLUSION CRITERIA** | | |
| --- | --- | --- |
|  | **No** | **Yes** |
| (1) Age ≥ 18 and ≤ 40 years |  | √ |
| (2) No present active medical, surgical and gynaecological problems |  | √ |
| (3) No present history of medical , surgical and gynecological problem/s |  | √ |
| (4) First elective LSC |  | √ |
| (7) Subject has given informed consent |  | √ |

| **SCREENING EXCLUSION CRITERIA** | | |
| --- | --- | --- |
|  | No | Yes |
| History of drug/alcohol abuse | √ |  |
| Patient taking warfarin or heparin | √ |  |
| Taking medication that affect wound healing, including glucocorticoid and immunosuppressive therapy | √ |  |
| Clinical relevant cardiovascular, gastrointestinal, hepatic, neurologic, endocrine, haematologic or other major systemic diseases making implementationof the protocol/intepretation of the result difficult. | √ |  |
| Mental condition rendering the subject unable to understand the nature, scope of the study | √ |  |
| Patient with chronic cough or other condition which may cause a rise in intra-abdominal pressure | √ |  |
| Presence of any congenital anterior abdominal wall defects | √ |  |
| Evidence of uncooperative attitude, including poor compliance including inability to attend follow-up visit | √ |  |

| **RELEVANT PREVIOUS & CONCOMITANT DISEASES / SURGERY** |
| --- |
| Are there any relevant previous or concomitant diseases and surgery  in the patient’s medical history?  If YES, please specify: ……………………………………………………………………………………………………. |
|  |

**VISIT: M**

**DATE: FTIME: H ID NO: H**

|  | **No** | **Yes** |
| --- | --- | --- |
| Did any undesirable drug effect occur? |  |  |
| If yes please specify | | |

**Has the subject been taking any other drug other than the drugs given for this study? If Yes, please provide detail in table below**

| **CONCURRENT MEDICATION** | | | | |
| --- | --- | --- | --- | --- |
| **MEDICATION**  *(Generic Name)* | **START DATE** | **STOP DATE** | **TOTAL DAILY DOSE**  *( Specify Unit)* | **REASON FOR USE** |
|  | **__ __, __ __,__ __**  dd mm yy | **__ __, __ __,__ __**  dd mm yy |  |  |
|  | **__ __, __ __,__ __**  dd mm yy | **__ __, __ __,__ __**  dd mm yy |  |  |
|  | **__ __, __ __,__ __**  dd mm yy | **__ __, __ __,__ __**  dd mm yy |  |  |

**1) POST OPERATIVE PAIN**

Tick the score:

| No  pain |  | Mild pain |  | Moderate |  | Severe |  | Very severe pain |  | Worst possible pain |
| --- | --- | --- | --- | --- | --- | --- | --- | --- | --- | --- |
| 0 | 1 | 2 | 3 | 4 | 5 | 6 | 7 | 8 | 9 | 10 |
|  |  |  |  |  |  |  |  |  |  |  |

**2) PHYSICAL EXAMINATION:**

BP:________________________PR:________________ T:__________________

| **SITE/SYSTEM** | **TICK ACCORDINGLY** | | **IF ‘ABNORMAL’, GIVE PERTINENT DETAIL** |
| --- | --- | --- | --- |
|  | **NORMAL** | **ABNORMAL** |  |
| Respiratory System |  |  |  |
| Cardiovascular System |  |  |  |
| Abdominal |  |  |  |

OTHER SYSTEMS:__________________________________________________________

**3) WOUND EVALUATION SCALE**

YES (1)	NO (0)

i. **STEPOFF BORDERS**
ii. **CONTOUR IRREGULARITIES**
iii. **SCAR WIDTH >2MM**
iv. **EDGES INVERSION(Sinking/Curling)**
v. **DISCHARGE**
vi. **OVERALL COSMESIS	ACCEPTABLE(1)	POOR(0)**

/6

**TOTAL MARKS:**

**4) COSMETIC APPEARANCE (VAS)**

**Poor 0 _____________________________________________________10cm	Very good**

**5) PATIENT SATISFACTION SCORE (PSS)**

| Not satisfied |  |  |  |  |  |  |  |  |  | Very satisfied |
| --- | --- | --- | --- | --- | --- | --- | --- | --- | --- | --- |
| **0** | **1** | **2** | **3** | **4** | **5** | **6** | **7** | **8** | **9** | **10** |
|  |  |  |  |  |  |  |  |  |  |  |

**6) BLOOD INVESTIGATIONS**

| **FBC** | | **RFT** | | **LFT** | |
| --- | --- | --- | --- | --- | --- |
| TWBC |  | Creatinine |  | ALT |  |
| Haemoglobin |  | Urea |  | AST |  |
| Platelet |  |  |  | Total protein |  |
|  |  |  |  | Albumin |  |

**Radiological Form:**

Visit 1 (Day 3 post operation)

A) Uterus Measurement

|  | 1^st^ reading | 2^nd^ reading | 3^rd^ reading | Average |
| --- | --- | --- | --- | --- |
| Transverse |  |  |  |  |
| Longitudinal |  |  |  |  |

B)

|  | Pulstility index (PI) | Resistive index (RI) | Percentage of the colour / power intensity |
| --- | --- | --- | --- |
| Caesarean uterine scar |  |  |  |
| Superficial caesarean skin wound |  |  |  |

C) Subcutaneous tissue thickness:____mm

Visit 2 (WEEK 2 post operation)

A) Uterus Measurement

|  | 1^st^ reading | 2^nd^ reading | 3^rd^ reading | Average |
| --- | --- | --- | --- | --- |
| Transverse |  |  |  |  |
| Longitudinal |  |  |  |  |

|  | Pulstility index (PI) | Resistive index (RI) | Percentage of the colour / power intensity |
| --- | --- | --- | --- |
| Caesarean uterine scar |  |  |  |
| Superficial caesarean skin wound |  |  |  |

C) Subcutaneous tissue thickness:____mm

Visit 3 (WEEK 4 post operation)

A) Uterus Measurement

|  | 1^st^ reading | 2^nd^ reading | 3^rd^ reading | Average |
| --- | --- | --- | --- | --- |
| Transverse |  |  |  |  |
| Longitudinal |  |  |  |  |

B)

|  | Pulstility index (PI) | Resistive index (RI) | Percentage of the colour / power intensity |
| --- | --- | --- | --- |
| Caesarean uterine scar |  |  |  |
| Superficial caesarean skin wound |  |  |  |

C) Subcutaneous tissue thickness:____mm

Visit 4 (WEEK 6 post operation)

A) Uterus Measurement

|  | 1^st^ reading | 2^nd^ reading | 3^rd^ reading | Average |
| --- | --- | --- | --- | --- |
| Transverse |  |  |  |  |
| Longitudinal |  |  |  |  |

B)

|  | Pulstility index (PI) | Resistive index (RI) | Percentage of the colour / power intensity |
| --- | --- | --- | --- |
| Caesarean uterine scar |  |  |  |
| Superficial caesarean skin wound |  |  |  |

C) Subcutaneous tissue thickness:____mm

**ADVERSE EVENT FORM**

Onset of adverse event:

Description of adverse event:

Severity	:	mild

moderate

severe

Relation to drug	:	probable

possible

unlikely

Outcome	:	recovered

stabilized

recovered with

sequelae

not yet recovered

died

unknown

**Consent Form (Malay Language)**

**MAKLUMAT KAJIAN**

**Tajuk Kajian:**	Kajian tentang kesan ekstrak ikan Haruan (Channa striatus) secara oral ke atas wanita selepas bersalin secara pembedahan Lower Segment Caesarean Section (LSCS)

**Nama Penyelidik:**	Prof Madya Dr Azidah Abdul Kadir (No MMC 33310)

Prof Dr Nik Hazlina Nik Hussain ( No MMC 29031)

Puan Siti Zubaidah Ab Wahab

Dr Mohd Rizal Abu Bakar

**Pengenalan**

Anda dipelawa untuk menyertai satu kajian penyelidikan secara sukarela yang melibatkan estrak ikan haruan ke atas wanita yang bersalin secara pembedahan Lower Segment Caesarean Section (LSCS). Sebelum anda bersetuju untuk menyertai kajian penyelidikan ini, adalah penting anda membaca dan memahami borang ini. Sekiranya anda menyertai kajian ini, anda akan menerima satu salinan borang ini untuk disimpan sebagai rekod anda.

Ikan Haruan atau nama saintifik Channa striatus, adalah sejenis ikan air tawar yang biasanya di makan di kawasan Asia Pasifik Selatan. Ikan haruan sudah dikenali sejak zaman berzaman sebagai salah satu sumber protein dan juga sebagai ubat tradisional. Ia dipercayai dapat mempercepatkan penyembuhan luka dan juga mengurangkan sakit selepas pembedahan. Ia dikenali sebagai salah satu sumber makanan yang berkhasiat terutama di kalangan masyarakat Asia. Ini adalah keranan ia mengandungi kandungan asid amino dan juga ‘fatty acids’. Ikan ini juga terkenal dikalangan wanita selepas bersalin kerana fungsinya untuk mempercepatkan pemyembuhan.

Banyak kajian saintifik telah dijalankan di Malaysia telah membongkar rahsia potensi perubatan ikan ini. Kajian yang dijalankan menunjukkan bahawa ikan ini mempunyai potensi sebagai ubat tahan sakit yang setara dengan morfin. Kesan ini menjadikannya sesuai sebagai penggunaan untuk mengurangkan kesakitan selepas pembedahan. Ia juga mempunyai kesan anti radang, anti bacteria dan juga anti kanser.

Penyertaan anda di dalam kajian ini dijangka mengambil masa sehingga 6 minggu. Seramai 84 pesakit akan menyertai kajian ini.

**Tujuan Kajian**

Kajian ini bertujuan adalah untuk mengkaji kesan ekstrak ikan haruan terhadap wanita selepas pembedahan kelahiran terhadap penyembuhan luka pembedahan, tahap kesakitan selepas pembedahan dan tahap keselamatan ekstrak.

**Kelayakan Penyertaan**

Doktor yang bertanggungjawab dalam kajian ini atau salah seorang kakitangan kajian telah membincangkan kelayakan untuk menyertai kajian ini dengan anda. Adalah penting anda berterus terang dengan doktor dan kakitangan tersebut tentang sejarah kesihatan anda. Anda tidak seharusnya menyertai kajian ini sekiranya anda tidak memenuhi semua syarat kelayakan.

Beberapa **keperluan** untuk menyertai kajian ini adalah –

- Anda mesti berumur diantara 18 dan 40 tahun
- Tiada penyakit yang aktif
- Anda menjalani pembedahan kelahiran yang pertama

Anda **tidak boleh** menyertai kajian ini sekiranya –

- Anda telah mengambil ubat-ubatan tradisional sekurang-kurangnya 3 bulan yang lalu.
- Mempunyai masalah alkohol atau dadah
- Mengambil ubat heparin atau warfarin
- Mempunyai penyakit jantung, hati, perut,hematologi atau penyakit sendi yang boleh mempengaruhi keputusan kajian.
- Anda mempunyai penyakit yang memerlukan anda mengambil steroid atau rawatan immunosupresif.
- Pesakit yang mempunyai batuk kronik
- Anda mempunyai penyakit kongenital bahagian dinding perut.
- Anda mempunyai penyakit mental yang menyebabkan anda tidak dapat memahami protokol kajian.

**Prosedur-prosedur Kajian**

Pada lawatan pertama anda, (iaitu sehari selepas pembedahan kelahiran) sekiranya anda setuju menyertai kajian, anda akan menjalani ujian fizikal, tanda-tanda vital dan juga dikehendaki mengisi soal selidik demografi. Anda juga akan diminta memberi maklumat tentang sejarah perubatan anda. Selepas itu 3 ml darah akan diambil untuk ujian fungsi hati, ginjal dan “full blood count” untuk tujuan profil keselamatan ekstrak.

Tiga hari selepas pembedahan keputusan ujian darah dilihat untuk mengesahkan kesesuaian untuk menyertai kajian ini. Kemudian, tahap kesakitan akan dianalisa dan luka pembedahan akan diperiksaan menggunakan “Wound Evaluation Scale (WES)”, “Visual Analogue Scale (VAS)” dan “Patient Satisfaction Score (PSS)”. Anda kemudian akan menjalani imbasan ultrasound. Kemudian anda akan disaran untuk mengambil sama ada ekstrak ikan haruan ataupun ubat placebo. Anda dikehendaki mengambil ubat tersebut sekali sehari selama 42 hari atau 6 minggu.

Semasa kajian ini, anda tidak dibenarkan mengambil sebarang ubat tradisional yang lain atau memakan ikan haruan segar atau dimasak. Pemeriksaan fizikal, pmeriksaan luka pembedahan (WES, VAS dan PSS) dan penilaian tahap kesakitan serta imbasan ultrasound akan dilakukan pada minggu ke-2, minggu ke-4 dan minggu ke-6 selepas pembedahan. Pada minggu ke-6, 5 ml darah akan diambil sekali lagi untuk tujuan profil keselamatan ektrak.

**Risiko**

Setakat ini tiada apa-apa risiko yang didapati. Jika apa-apa maklumat penting yang baru dijumpai semasa kajian ini yang mungkin mengubah persetujuan anda untuk terus menyertai kajian ini, anda akan diberitahu secepat mungkin.

**Melaporkan Pengalaman Kesihatan**

Jika anda mengalami apa-apa kecederaan, kesan buruk, atau apa-apa pengalaman kesihatan yang luarbiasa semasa kajian ini, pastikan anda memberitahu penyelidik iaitu Siti Zubaidah Ab Wahab di talian 017-9859795 atau Dr Azidah Abdul Kador di talian 012-9286006 secepat mungkin. Anda boleh membuat panggilan pada bila-bila masa, siang atau malam, untuk melaporkan pengalaman sedemikian.

**Penyertaan Dalam Kajian**

Penyertaan anda dalam kajian ini adalah secara sukarela. Anda boleh menolak untuk menyertai kajian ini atau anda boleh menamatkan penyertaan anda pada bila-bila masa, tanpa sebarang hukuman atau kehilangan manfaat yang sepatutnya anda perolehi.

Penyertaan anda juga mungkin boleh diberhentikan oleh doktor yang terlibat dalam kajian ini tanpa persetujuan anda. Sekiranya anda berhenti menyertai kajian ini, doktor yang terlibat di dalam kajian ini atau salah seorang kakitangan akan berbincang dengan anda mengenai apa-apa isu perubatan berkenaan dengan pemberhentian penyertaan anda.

**Manfaat yang Mungkin**

Prosedur kajian ini akan diberikan kepada anda tanpa kos. Anda mungkin menerima maklumat tentang kesihatan anda daripada pemeriksaan fizikal. Hasil atau maklumat kajian ini diharapkan, dapat memberi manfaat kepada pesakit-pesakit pada masa hadapan.

**Soalan**

Sekiranya anda mempunyai sebarang soalan mengenai prosedur kajian ini atau hak-hak anda, sila hubungi;

Siti Zubaidah Ab Wahab

Jabatan Perubatan Keluarga

Pusat Pengajian Sains Perubatan

Kampus Kesihatan

Universiti Sains Malaysia

16150, Kubang Kerian

Kelantan

No Tel: 017-9859795

Dr Azidah Abdul Kadir (No MMC: 33310)

Jabatan Perubatan Keluarga

Pusat Pengajian Sains Perubatan

Kampus Kesihatan

Universiti Sains Malaysia

16150, Kubang Kerian

Kelantan

No tel: 012-9286006/09-7676604/09-7676608

Sekiranya anda mempunyai sebarang soalan berkaitan kelulusan etika kajian ini, sila hubungi;

Puan Mazlita Zainal Abidin

Setiausaha Jawatankuasa Etika Penyelidikan (Manusia) USM

Pelantar Penyelidikan Sains Klinikal, USM Kampus Kesihatan.

No. Tel: 09-7672355 / 09-7672352

**Kerahsiaan**

Maklumat perubatan anda akan dirahsiakan oleh doktor dan kakitangan kajian. Ianya tidak akan dedahkan secara umum melainkan jika ia dikehendaki oleh undang-undang. Data yang diperolehi dari kajian yang tidak mengenalpasti anda secara perseorangan mungkin akan diterbitkan untuk tujuan memberi pengetahuan baru. Rekod perubatan anda yang asal mungkin akan dilihat oleh pihak penyelidik, Lembaga Etika kajian ini dan pihak berkuasa regulatori untuk tujuan mengesahkan prosedur dan/atau data kajian klinikal. Maklumat perubatan anda mungkin akan disimpan dalam komputer dan diproses dengannya. Dengan menandatangani borang persetujuan ini, anda membenarkan penelitian rekod, penyimpanan maklumat dan pemindahan data seperti yang dihuraikan di atas.

**Tandatangan**

Untuk dimasukkan ke dalam kajian ini, anda atau wakil sah anda mesti menandatangani serta mencatatkan tarikh halaman tandatangan.

**BORANG KEIZINAN PESAKIT/ SUBJEK (HALAMAN TANDATANGAN)**

***Tajuk Kajian:***	Kajian mengenai kesan ikan Haruan (Channa striatus) ke atas wanita selepas pembedahan

***Nama Penyelidik:***	Dr Azidah Abdul Kadir ( No MMC 33310)

Siti Zubaidah Binti Ab Wahab

Untuk menyertai kajian ini, anda atau wakil sah anda mesti menandatangani mukasurat ini. Dengan menandatangani mukasurat ini, saya mengesahkan yang berikut:

- Saya telah membaca semua maklumat dalam Borang Maklumat dan Keizinan Pesakit ini termasuk apa-apa maklumat berkaitan risiko yang ada dalam kajian dan saya telah pun diberi masa yang mencukupi untuk mempertimbangkan maklumat tersebut.
- Semua soalan-soalan saya telah dijawab dengan memuaskan.
- Saya, secara sukarela, bersetuju menyertai kajian penyelidikan ini, mematuhi segala prosedur kajian dan memberi maklumat yang diperlukan kepada doktor, para jururawat dan juga kakitangan lain yang berkaitan apabila diminta.
- Saya boleh menamatkan penyertaan saya dalam kajian ini pada bila-bila masa.
- Saya telah pun menerima satu salinan Borang Maklumat dan Keizinan Pesakit untuk simpanan peribadi saya.

Nama Pesakit (Dicetak atau Ditaip)	Nama Singkatan & No. Pesakit

No. Kad Pengenalan Pesakit (Baru)	No. K/P (Lama)

Tandatangan Pesakit atau Wakil Sah	Tarikh (dd/MM/yy)

(Masa jika perlu)

Nama & Tandatangan Individu yang Mengendalikan	Tarikh (dd/MM/yy)

Perbincangan Keizinan (Dicetak atau Ditaip)

Nama Saksi dan Tandatangan	Tarikh (dd/MM/yy)

Nota:	i) Semua subjek/pesakit yang mengambil bahagian dalam projek penyelidikan ini tidak dilindungi insuran.
